# Assessment of Changes in the Expression of Genes Involved in Insulin Signaling and Glucose Transport in Leukocytes of Women with Gestational Diabetes During Pregnancy and in the Postpartum Period

**DOI:** 10.3390/ijms252313094

**Published:** 2024-12-05

**Authors:** Andrzej Zieleniak, Monika Zurawska-Klis, Karolina Laszcz, Krystsina Bulash, Dagmara Pacyga, Katarzyna Cypryk, Lucyna Wozniak, Marzena Wojcik

**Affiliations:** 1 Department of Structural Biology, Faculty of Biomedical Sciences, Medical University of Lodz, 90-752 Lodz, Poland; andrzej.zieleniak@umed.lodz.pl (A.Z.); lucyna.wozniak@umed.lodz.pl (L.W.); 2 Department of Internal Diseases and Diabetology, Medical University of Lodz, 92-213 Lodz, Poland; monika.zurawska-klis@umed.lodz.pl; 3 Faculty of Biomedical Sciences, Medical University of Lodz, 90-752 Lodz, Poland; karolina.laszcz@student.umed.lodz.pl (K.L.); krystsina.bulash@student.umed.lodz.pl (K.B.); dagmara.pacyga@student.umed.lodz.pl (D.P.)

**Keywords:** gestational diabetes mellitus (GDM), glucose transporters, insulin signaling, leukocytes

## Abstract

Not much is currently known about disturbances in insulin signaling and glucose transport in leukocytes of women with gestational diabetes mellitus (GDM) during and after pregnancy. In this study, the expression of insulin signaling (*INSR*, *IRS1*, *IRS2* and *PIK3R1)-* and glucose transporter (*SLC2A1*, *SLC2A3* and *SLC2A4*)-related genes in the leukocytes of 92 pregnant women was assayed using quantitative RT-PCR. The cohort consisted of 44 women without GDM (NGT group) and 48 with GDM (GDM group) at 24–28 weeks of gestation. GDM women were then tested again one year after childbirth (pGDM group: 14 women (29.2%) with abnormal glucose tolerance (AGT) and 34 women (70.8%) with normoglycemia). The GDM and NGT groups were closely matched for gestational age and parameters of obesity, such as pre-pregnancy body mass index (BMI), pregnancy weight, and gestational weight gain (GWG) (*p* > 0.05). Compared to the NGT group, the GDM and pGDM groups were hyperglycemic, but the GDM group featured a more highly insulin-resistant condition than the pGDM group, as reflected by higher fasting insulin (FI) levels and the values of the homeostasis model assessment for insulin resistance (HOMA-IR) (*p* < 0.05). In leukocytes from the GDM and pGDM groups, *PIK3R1*, *SLC2A1*, and *SLC2A3* were upregulated and *IRS1* was downregulated, with a larger magnitude in fold change (FC) values for *PIK3R1* and *IRS1* in the GDM group and for *SLC2A1* and *SLC2A3* in the pGDM group. The expression of *SLC2A4* was unchanged in the GDM group but upregulated in the pGDM group, where it was inversely correlated with HOMA-IR (*rho* = −0.48; *p* = 0.007). Although the *INSR* and *IRS2* levels did not significantly differ between the groups, the *IRS2* transcript positively correlated with pregnancy weight, fasting plasma glucose, FI, and HOMA-IR in the GDM group. Our findings indicate that pronounced quantitative changes exist between the GDM and pGDM groups with respect to the expression of certain genes engaged in insulin signaling and glucose transport in leukocytes, with insulin resistance of a variable degree. These data also highlight the relationship of leukocyte *SLC2A4* expression with insulin resistance in the postpartum period.

## 1. Introduction

Gestational diabetes mellitus (GDM), defined as a carbohydrate intolerance with onset or first recognition during pregnancy, is one of the most common metabolic disorders among pregnant women, affecting 16.7% of all pregnancies, as reported in 2021 by the International Diabetes Federation (IDF). In Poland, its prevalence is estimated to be 9.7% [1]. Of note is that the incidence of GDM has been on the rise, mainly because of increasing rates of maternal overweight/obesity and advancing maternal age [2]. Importantly, GDM constitutes a significant public health and clinical problem as it is related to adverse outcomes for both mother and offspring. For the mother, GDM is associated with gestational hypertension, caesarean section, and pre-eclampsia during pregnancy, and significantly increased risks of abnormal glucose tolerance (AGT) and cardiovascular disease after pregnancy [3]. For children born to mothers with GDM, there are increased rates of obesity and an increased cardiometabolic risk in both childhood and adulthood [3].

The exact pathogenesis of GDM is still debated; however, insulin resistance, defined as an impaired response of target cells to insulin, is thought to be a key pathological feature of diabetic pregnancy, similarly to type 2 diabetes mellitus (T2DM) [4]. Although the underlying molecular mechanisms of insulin resistance in GDM are complex and still not fully understood, multiple defects in the insulin signaling pathway have been shown to be responsible for impaired glucose metabolism in target tissues of diabetic pregnancies with features of insulin resistance [4,5,6]. In keeping with this, alterations at several levels of the insulin-signaling cascade and in glucose transport have been identified in subcutaneous adipose tissue and skeletal muscle from insulin-controlled GDM patients in non-obese and obese cohorts, suggesting that both GDM and obesity can impact on components of the insulin signaling pathway, leading to impaired glucose transport in these tissues [6]. In the placenta, although post-receptor defects have been recognized in the insulin signaling pathway in non-obese and obese women with insulin-controlled GDM, a controversy still exists about changes in the expression of placental glucose transporter isoforms 1 (insulin-independent GLUT1), 3 (GLUT3 being a high-affinity glucose transporter), and 4 (insulin-dependent GLUT4), encoded by the *SLC2A1*, *SLC2A3*, and *SLC2A4* genes, respectively, and their roles in disturbances in transplacental energy substrate supply in diabetic pregnancy [5,7,8,9,10].

Leukocytes are key effectors of the immune response against invading pathogens, as well as central mediators of inflammation, which has been found in GDM patients during and after pregnancy [11,12]. Moreover, these cells can reflect pathological changes elsewhere in the body, as has been implied in the literature [13]. Hence, leukocytes have gained importance as a potential cellular model for investigating numerous molecular mechanisms linked to the pathophysiology of GDM and its transition to postpartum AGT [12,14,15], overcoming the problem of metabolic tissues not being easily sampled from pregnant women. Despite this, data about molecular disturbances in the insulin signal transduction pathway, as well as the expression of glucose transporters in leukocytes of women with GDM during and after pregnancy, are very poor.

This study aims to (i) assess metabolic changes in pregnant women with GDM at the time of GDM diagnosis (24–28 weeks of gestation) and 1 year after delivery; (ii) quantify changes in the mRNA levels of a cluster of genes involved in the insulin signaling pathway [i.e., insulin receptor (*INSR*), insulin receptor substrates 1 and 2 (*IRS1* and *IRS2*), and p85α phosphatidylinositol 3-kinase (*PIK3R1*)] and glucose transport (i.e., *SLC2A1*, *SLC2A3*, and *SLC2A4*) in the leukocytes of the women during and after diabetic pregnancy; (iii) look for associations between the expression of the aforementioned genes and clinical phenotypes of the patients in both periods examined; and finally, (iv) evaluate the usefulness of the studied transcripts and clinical parameters as potential predictors of postpartum AGT in the women with GDM.

## 2. Results

### 2.1. Comparison of Clinical Phenotypes of Diabetic Patients at GDM Diagnosis and 1 Year Postpartum

The clinical characteristics of the GDM patients at the time of GDM diagnosis and 1 year postpartum are listed in Table 1 and Table 2, respectively. The GDM and NGT groups were similar in terms of gestational age and parameters of obesity, such as pre-pregnancy body mass index (BMI), pregnancy weight, and gestational weight gain (GWG) (*p* > 0.05). Compared to women with normal pregnancy, those with GDM were slightly older (29.00 (26.00–32.00) vs. 32.00 (29.00–35.00), *p* = 0.014) and had significantly higher fasting plasma glucose (FPG; fold change (FC) = 1.07), 1 and 2 h plasma glucose concentrations (1 h-PG; FC = 1.20 and 2 h-PG; FC = 1.13, respectively), fasting insulin (FI; FC = 1.82), and values of the homeostasis model assessment for insulin resistance (HOMA-IR) (FC = 1.96; *p* < 0.001 for all the aforementioned variables), even after adjusting for maternal age (Table S1). There were no significant differences in glycated hemoglobin A1C (HbA1C), the HOMA-β cell function (HOMA-B) index, lipid profile [i.e., total cholesterol (TC), low-density lipoprotein (LDL-C), high-density lipoprotein (HDL-C) and triglycerides (TGs)] or C-reactive protein (CRP) between the GDM and NGT groups (*p* > 0.05; Table 2).

At the first-year follow-up, 14 (29.2%) of the 48 women in the GDM group had developed AGT. In particular, 13 (27.1%) women had prediabetes and only 1 (2.1%) had T2DM. Other than FPG and 2 h-PG, there were no significant differences in maternal characteristics between the patients with postpartum AGT vs. those with postpartum normal glucose tolerance, which might be due to the relatively small sample size. Therefore, for all statistical analyses, the pGDM group was used as a whole. As shown in Table 2, compared to the GDM and NGT groups, the pGDM group had significantly higher FPG and lower 2 h-PG, all the blood lipid parameters and CRP. The pGDM group also displayed significantly lower FI (FC = 0.77; *p* = 0.001), HOMA-IR (FC = 0.78; *p* = 0.005) and HOMA-B (FC = 0.69; *p* < 0.001) values than the GDM group; however, when these parameters were compared between the pGDM and NGT groups, FI (FC = 1.40; *p* = 0.005) and HOMA-IR (FC = 1.52; *p* = 0.001) remained elevated in the pGDM group while HOMA-B did not differ between the groups (*p* > 0.05). Of note is that postpartum BMI returned to that before pregnancy and postpartum HbA1C remained unchanged relative to the levels of the GDM and NGT groups during pregnancy (*p* > 0.05; Table 2).

### 2.2. Longitudinal Changes in the Transcriptional Levels of the Studied Genes

Table 3 shows the quantitative RT-PCR expression data for all the investigated genes during pregnancy and at the 1-year follow-up. During pregnancy, we found significant increases in the mRNA levels of *PIK3R1* (FC = 7.57; *p* < 0.001), *SLC2A1* (FC = 1.61; *p* < 0.001), and *SLC2A3* (FC = 4.01; *p* < 0.001), and a decrease in the expression of *IRS1* (FC = 0.23; *p* = 0.046) in the GDM group vs. the NGT group. No significant differences were seen in the mRNA expression of *INSR*, *IRS2*, or *SLC2A4* between the two groups. At the first year after delivery, the expression of *PIK3R1* (FC = 4.81; *p* < 0.001), *SLC2A1* (FC = 2.32; *p* < 0.001), *SLC2A3* (FC = 5.51; *p* < 0.001), and *SLC2A4* (FC = 2.46; *p* = 0.002) was significantly increased in the pGDM group with respect to the NGT group, and these changes were accompanied by IRS1 downregulation (FC = 0.42; *p* = 0.030). The *INSR* mRNA level was unaltered between the pGDM and NGT groups.

Of note is that the between-group differences in the expression of the investigated genes persisted after adjusting for maternal age (Table S2).

### 2.3. Correlations Between Clinical Phenotypes of Patients and the Transcriptional Levels of the Studied Genes

Spearman’s correlation test with multiple testing corrections using the false discovery rate (FRD) method was applied to detect relationships between the expression levels of the investigated genes and the clinical phenotypes of patients in the separate groups (NGT, GDM, and pGDM). The results of these analyses are provided in Table 4 and Table 5. There was no significant correlation between the mRNA expression of any of the genes studied and individual clinical parameters in the NGT group (Table S3). However, we noticed significant positive associations of the *IRS2* transcript with pregnancy weight (*rho* = 0.46; *p* = 0.013), FPG (*rho* = 0.42; *p* = 0.023), FI (*rho* = 0.44; *p* = 0.018), and HOMA-IR (*rho* = 0.48; *p* = 0.008) in the GDM group, and an inverse correlation of the *SLC2A4* transcript with HOMA-IR (*rho* = −0.48; *p* = 0.007) in the pGDM group.

### 2.4. The Potential of the Studied Transcripts and Clinical Parameters as Predictors for pAGT

**Figure 1:**
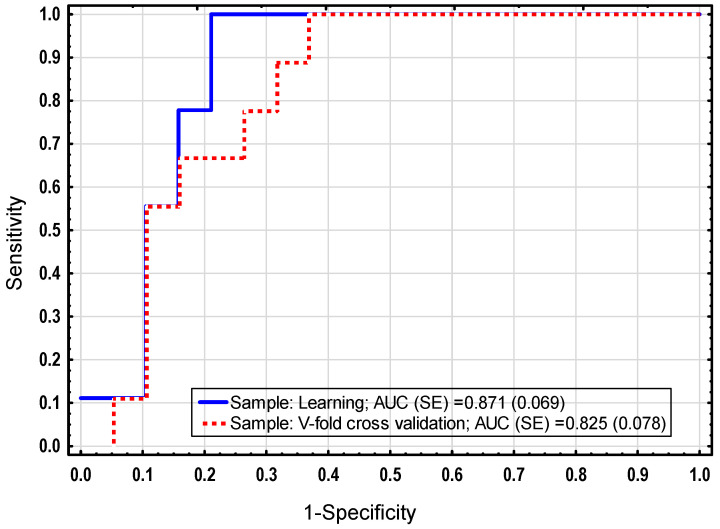
Predictive accuracy of logistic regression model based on pregnancy *INSR* expression and body weight for postpartum AGT. The presented ROC curves reflect performance of the model on learning and validation datasets.

To explore the predictive ability of the clinical parameters and expression profiles of the studied genes in identifying the pAGT group, we carried out receiver operating characteristic (ROC) curve analyses. As shown in Table 6, the largest areas under the ROC curves (AUCs) of individual variables were obtained using *INSR* (0.768), *IRS1* (0.705), pregnancy weight (0.705), pre-pregnancy BMI (0.660), *SLC2A3* (0.650), FPG (0.646), 1 h-PG (0.634), *IRS2* (0.626), HbA1C (0.617), and *PIK3R1* (0.615); however, among the aforementioned variables, only *INSR* and pregnancy weight achieved statistically significant discriminative power (*p* = 0.003 and *p* = 0.030, respectively), with optimal cut-off points of 0.66 and 82.90, respectively, for predicting any kind of postpartum glucose disturbance. We further analyzed multiple logistic regression models, incorporating clinical and expression data selected from the previous ROC analysis. Each model was built with the use of forward or backward stepwise selection of the prediction factors and validated using a V-fold cross-validation procedure. The resulting models were assessed by ROC analysis of their outcome for cross-validation datasets, where those with the greatest AUC were recognized as robust and reliable (not over-fitted). Of the different models generated, the most accurate one was identified [composed of *INSR* expression (OR = 11.6) and pregnancy body weight (OR = 1.1)], and its parameters are presented in Table 7. The postpartum AGT prediction model was characterized by the largest AUC for both learning and validation data, and its fitting was high in the cross-validation sample (AUC= 0.825, Figure 1), indicating its better discriminative performance than any of the single raw variable predictors. The risk model for the occurrence of pAGT was positively associated with gestational *INSR* expression and body weight.

## 3. Discussion

The present longitudinal study documents for the first time that in leukocytes of women with GDM during and after pregnancy, there are quantitative changes in the expression profiles of several important genes engaged in insulin signaling and glucose transport and, moreover, the mRNA level of one of the investigated genes, i.e., *SLC2A4*, is inversely correlated with HOMA-IR in the postpartum period. This study also provides important information about a potential clinical model for the prediction of postpartum AGT among women with GDM that includes pregnancy *INSR* expression and body weight.

From a clinical point of view, the GDM group in our study was characterized by hyperglycemia and hyperinsulinemia, reflecting an increase in insulin resistance, as assessed by HOMA-IR in this group. This agrees with the results of previous studies [16,17,18,19]. At the 1-year follow-up, the women with prior GDM had hyperglycemia, as evidenced by significantly higher FPG levels than during diabetic pregnancy. This suggests that a history of GDM has a detrimental effect on glucose homeostasis in the first year after delivery. Interestingly, this change was accompanied by reduced insulin resistance relative to the diabetic state. The significance of this is not obvious, although the weakened insulin resistance in the postpartum period might be due to the fact that the pGDM women returned to their pre-pregnancy BMI < 25 kg/m^2^. Changes in insulin resistance are known to be partly related to alterations in maternal fat mass during and after diabetic pregnancy [20]. Alternatively, the drop in postpartum insulin resistance might result from the weight-loss-associated attenuation of the inflammatory state, as CRP levels in the pGDM women were significantly decreased compared to those found during pregnancy with and without GDM. CRP is an acute-phase reactant that is widely used as a biomarker of inflammation [21], and previous evidence exists in support of its linkage to adiposity and insulin resistance in metabolic disorders [22,23]; however, it is unknown whether changes in postpartum BMI might contribute to alterations in circulating CRP levels and HOMA-IR values in women with GDM in the first year after delivery, and therefore further research is needed to verify this hypothesis.

The dysregulation of the insulin signaling pathway in insulin-dependent tissues such as skeletal muscle and adipose tissue is of critical importance in the development of insulin resistance and blood glucose homeostasis in GDM and T2DM, as outlined in many previous reports [6,24]. Interest in leukocytes has seen a resurgence in recent years, as investigators have unraveled the effect of metabolic regulation on these cells [25]. As such, we used leukocytes as a cellular model for analyzing alterations in the expression of genes related to insulin signaling, such as *INSR*, *IRS1*, *IRS2,* and *PIK3R1,* and those related to glucose transport, such as *SLC2A1*, *SLC2A3* and *SLC2A4,* in women with GDM at the time of GDM diagnosis and 1-year postpartum.

*INSR* encodes an insulin receptor that plays a key role in signal transmission between circulating insulin and the intracellular environment. To date, different levels of *INSR* expression have been reported in several physiologic and pathologic states, including diabetes, depending on tissue type and environmental perturbations [26]. In our study, leukocyte *INSR* expression was not significantly different in non-obese women with GDM during and after pregnancy, although large individual variations were seen. A similar observation has been made in muscle and adipose tissues of non-obese and obese women with insulin-controlled GDM vs. normal control women, as well as in skeletal muscle biopsies from T2DM diabetic and non-diabetic obese subjects after an overnight fast or a hyperinsulinaemic clamp [6,27,28]. Given the fact that all the pregnant women in our study were analyzed at the point of GDM diagnosis, i.e., before receiving medication, a question remains about whether insulin treatment in these patients could affect their leukocyte *INSR* gene expression? This possibility deserves further investigation.

The *IRS1* and *IRS2* genes encode IRS1 and IRS2 proteins, respectively, which are engaged in transmitting the signal between the insulin receptor and phosphoinositide 3-kinase (PI3K). The p85 regulatory subunit plays a crucial role in this process and then affects the induction of end-point events, such as GLUT4 translocation to the cell membrane and glucose uptake into muscle cells and adipocytes [29]. In this study, leukocyte *IRS1* mRNA levels were markedly reduced in the GDM group (FC = 0.23; *p* = 0.046) and pGDM group (FC = 0.42; *p* = 0.030) compared to the NGT group; however, the magnitude of this decrease was greater in the GDM group, which was more insulin-resistant than the pGDM group. Thus, these findings show that leukocyte IRS1 gene expression attenuates as insulin resistance decreases from pregnancy to postpartum in women with GDM, suggesting that leukocyte *IRS1* transcript level could be related to whole-body insulin resistance status in GDM women at the time of GDM diagnosis and at 1-year follow-up. Although we did not find any correlation between the degree of *IRS1* gene downregulation and HOMA-IR value in either the GDM or pGDM patients, there is strong evidence from genetically engineered mouse models of *IRS1* deficiency and overexpression that IRS1 is directly linked to insulin resistance [30,31]. Furthermore, several *IRS1* polymorphisms have been shown to be associated with insulin resistance and GDM in some populations [32,33,34]. Interestingly, the GDM-related decrease in leukocyte *IRS1* gene expression observed in our study coincides with the results of a previous study showing IRS1 downregulation in adipose tissue and skeletal muscle from non-obese pregnancies complicated by GDM, suggesting that this change may not be unique to leukocytes in GDM [6]. With respect to IRS2, we found that its expression pattern was similar in the leukocytes of the GDM women during and after pregnancy, consistent with what has previously been shown in leukocytes of individuals with insulin resistance [35]. Nevertheless, leukocyte *IRS2* expression correlated significantly with pregnancy weight, FPG, FI, and HOMA-IR in the GDM subjects, pointing to a linkage of this molecule with adiposity, glucose metabolism, and insulin resistance during diabetic pregnancy. Although accumulating evidence from animal studies indicates that loss of the Irs2 gene leads to diabetes in mice due to a failure in B-cell function and peripheral and hepatic insulin resistance [36,37], the role of IRS2 in the pathophysiology of human diabetes is not completely understood, and different gene expression patterns have been observed in diabetic patients depending of cell type, patients’ characteristics, and the epigenetic mechanisms involved [6,38,39]. Thus, additional work is needed to untangle this complex issue not only in leukocytes, but also in insulin-sensitive tissues of diabetic patients.

The *PIK3R1* gene encodes a p85 regulatory subunit of PI3K, i.e., an essential component of the PI3K enzyme that mediates insulin’s metabolic actions, and its dysregulation has been implicated in the pathophysiology of insulin resistance and diabetes [40,41,42]. Furthermore, recent studies have highlighted the association of *PI3KR1* gene polymorphism with GDM [43] and T2DM [44]. The present study demonstrated that leukocyte *PIK3R1* expression was upregulated in the GDM and pGDM groups compared to the NGT group, although to a different extent. In the GDM group, which displayed a higher HOMA-IR value than the pGDM group, the increase in *PIK3R1* level was greater (FC = 7.57; *p* < 0.001) than in the pGDM group (FC = 4.81; *p* < 0.001). These data thus suggest that quantitative changes in leukocyte *PIK3R1* expression between the GDM and pGDM groups may be linked to alterations in the insulin-resistant states of both groups, although no significant correlations were found between the *PIK3R1* levels and HOMA-IR values in the GDM and pGDM groups. This interpretation is supported by previous studies on mouse models of gestational diabetes where increased PI3Kp85α expression levels were observed to be associated with insulin resistance [41]. Furthermore, an increased abundance of *PIK3R1* mRNA has also been found in insulin-resistant subjects with T2DM [45]. Although the exact molecular details linking PI3Kp85α overexpression and insulin resistance are still debated, the overexpression of PI3Kp85α has been proposed to impair signal transmission and cause insulin resistance by disrupting the activity of the p85α/catalytic subunit (p110) complex of PI3K and the connection between PI3K and IRS1 [41,46]. However, nothing is reported regarding this phenomenon in human leukocytes under diabetic conditions, emphasizing the need for further studies in this field.

An increasing number of studies have aimed to link GLUT1, GLUT3, and GLUT4 dysregulation to diabetes pathology, but results remain conflicting, likely due to differences in cell types used, patient characteristics—especially the distinct degree of obesity and different methods of treatment—and patient diagnostic criteria and thresholds of glycemic control with respect to GDM patients [5,6,7,8,9,10,35,47]. Out of the aforementioned GLUTs, the insulin-responsive facilitative glucose transporter GLUT4 has been acknowledged as a major transporter isoform in maintaining whole-body glucose homeostasis, and its presence has been well documented in the muscles, heart, and adipose tissue [48]. Despite existing evidence that GLUT4 is expressed in peripheral blood leukocytes [49,50], there are no studies evaluating leukocyte GLUT4 expression in patients with GDM during and after pregnancy. In the present study, we observed the lack of a significant change in leukocyte GLUT4 mRNA expression between the GDM and NGT groups, similarly to the results of Kipmen-Korgun et al. [50], who reported unchanged *SLC2A4* expression in leukocytes of T2DM patients. Interestingly, the postpartum gene expression of GLUT4 was upregulated in our study, and its level was inversely correlated with maternal HOMA-IR in the pGDM group, implying that the improvement in insulin sensitivity postpartum in the women with prior GDM could be partially due to *SLC2A4* upregulation. With respect to GLUT1 and GLUT3, we found that their mRNA levels increased from pregnancy to postpartum in the women with GDM, and the increases observed for GLUT3 in the GDM and pGDM groups were of greater magnitude than those detected for GLUT1 in both groups. Although the quantitative significance of this latter effect in the GDM and pGDM groups remains to be further explored, the expression of the GLUT3 transporter appears to be predominant among the studied GLUT isoforms in the leukocytes of diabetic women at the time of GDM diagnosis and 1-year postpartum. This is consistent with a previous report suggesting the importance of GLUT3 upregulation in placental glucose uptake in GDM by regulating hyperglycemia [51]. Considering the aforementioned data and the fact that GLUT3 has a much higher glucose affinity than GLUT4 [52], we cannot rule out the possibility that the observed GLUT3 overexpression, along with GLUT1 upregulation, in the leukocytes of the women with GDM could represent a compensatory mechanism to maintain glucose influx to the leukocytes despite GLUT4 being lost in these cells. This hypothesis remains to be further explored, but it is interesting to note that we did not identify significant correlations between changes in leukocyte GLUT 3 and GLUT 1 transcripts and metabolic parameters, either during diabetic pregnancy or the postpartum period. This suggests that leukocyte GLUT1 and GLUT3 gene expression might be influenced by other GDM- and postpartum-related conditions not assessed in this study.

Currently, transcriptomics is considered as a valuable molecular tool to better understand the pathophysiology of the transition from GDM to postpartum AGT [12]. Therefore, our intention was also to develop a model using the expression and clinical data to predict the risk of developing postpartum AGT occurring in the first year after delivery among the women with a history of GDM. Of the different logistic regression models constructed in our study, the model including a mix of two variables, i.e., expression and pregnancy body weight, achieved a good predictive performance (AUC = 0.825), making it the choice for external validation in a larger cohort in the future.

This study has several strengths and limitations. A strength of the study is its longitudinal nature with detailed metabolic profiles of the patients during and after diabetic pregnancy, who were selected based on strict inclusion and exclusion criteria and whose pre-pregnancy BMIs and gestational ages were comparable, thus reducing the interference of confounders on the observed results. Nonetheless, there are several limitations in our study. Firstly, there was the lack of a group of women after childbirth without a history of GDM, since the participation of healthy pregnant women in postpartum OGTT was entirely voluntary and they were not interested in returning to the clinic in the first year after giving birth to perform this test. Secondly, we did not separate leukocytes into subsets because our study intended to establish overall gene expression alterations in the systemic circulation of the diabetic pregnant women at their diagnosis and at 1-year postpartum. As such, future work would be immensely beneficial in understanding potential leukocyte cell subset-specific gene expression changes during and after diabetic pregnancy. Finally, we did not have information on the diet and physical activity of the patients, and whether these factors could influence changes in the expression of the genes examined is subject to further investigation. However, despite the limitations mentioned, our study provides data indicating that insulin signaling and glucose transport in peripheral blood leukocytes are apparently influenced by GDM and the postpartum period, at least in part at the transcriptional level, thus expanding the concept of the dysfunction of insulin-sensitive tissues during these two periods to systemic circulation. Specifically, there were quantitative differences between the expressions of *IRS1*, *PIK3R1*, *SLC2A1*, *SLC2A3,* and *SLC2A4* among the patients with GDM during pregnancy and at 1-year postpartum, i.e., in the two studied groups differing in the degree of insulin resistance. The present study is also worthwhile as it provides an important insight into the association between leukocyte GLUT4 gene expression and insulin resistance in the postpartum period, as such information is lacking in the literature. Further work is therefore required to determine the functional significance of this relationship. Our study also confirms the generally accepted fact that women in whom a previous pregnancy was complicated by GDM are at high risk of developing AGT later in life, and furthermore provides a model for predicting postpartum AGT in women with GDM; however, future studies with independent external validation are needed to confirm our findings and the relevance of this model for clinical application.

## 4. Materials and Methods

### 4.1. Study Design and Participants

**Figure 2:**
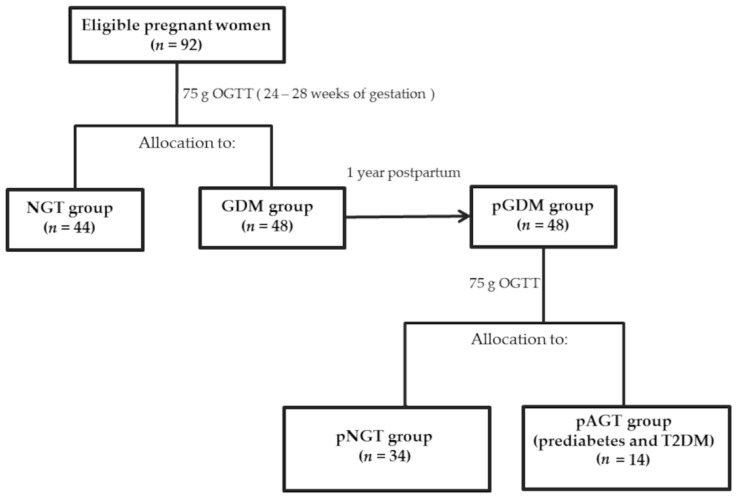
Allocation of patients to appropriate study groups at GDM diagnosis and 1 year after childbirth based on OGTT measurements.

This was a longitudinal study including 48 pregnant women with GDM during pregnancy (i.e., GDM group) and at 1-year postpartum (i.e., pGDM group) and 44 healthy pregnant women (i.e., NGT group), who were examined at the Outpatient Department of Diabetology, Lodz, Poland, and who were willing to participate in the study. All pregnant women (*n* = 92) were enrolled between 24 and 28 weeks of gestation, or later if it was not possible during this period (median, 27.5 weeks of gestation [interquartile range (IQR), 27 to 29]), and underwent a 75 g oral glucose tolerance test (OGTT). The status of GDM was assessed according to the World Health Organization (WHO) 2013/International Association of Diabetes in Pregnancy Study Groups (IADPSG) 2010 recommendations [53,54], as detailed below. The women with GDM (*n* = 48) had a further OGTT one-year postpartum, and based on the Polish Diabetes Association (PDA) criteria [55], they were classified as having normal glucose tolerance (*n* = 34) or AGT (*n* = 14). Of the women with AGT, 13 had prediabetes [i.e., impaired fasting glucose (IFG) or impaired glucose tolerance (IGT)] and 1 had T2DM. The procedure for assigning patients to the appropriate study groups is shown in Figure 2.

The inclusion criteria were as follows: (1) diagnosis of GDM according the WHO 2013/IADPSG 2010 guidelines [53,54]; (2) Caucasian ethnicity; (3) singleton pregnancy; and (4) maternal age between 18 and 40 years. The exclusion criteria were as follows: (1) family history of diabetes in first-degree relatives; (2) GDM in a previous pregnancy; and (3) autoimmune diseases, infectious diseases, or endocrine disorders, including polycystic ovary syndrome (PCOS). All patients were nonsmokers by self-report.

The study was conducted in accordance with the Declaration of Helsinki and approved by the Ethical Committee of the Medical University of Lodz (No. RNN/676/14/KB from 23 September 2014).

### 4.2. Diagnosis of GDM and Postpartum OGTT Diagnostic Categories

GDM was diagnosed if the woman had at least one of the following plasma glucose values from the 75 g OGTT: (equal to or exceeding) fasting 92 mg/dL (5.1 mmol/L), 1 h 180 mg/dL (10.0 mmol/L), and 2 h 153 mg/dL (8.5 mmol/L) [53,54]. Pregnant women who reached these thresholds were included in the GDM group; otherwise, they were allocated to the NGT group.

Postpartum AGT was evaluated based on the 75 g OGTT, as follows [55]: IFG [(FPG between 100 mg/dL (5.6 mmol/L) and 125 mg/dL (6.9 mmol/L)], IGT [2 h-PG between 140 mg/dL (7.8 mmol/L) and 199 mg/dL (11.1 mmol/L)], and T2DM [FPG ≥ 126 mg/dL (≥7.0 mmol/L) and/or 2 h-PG ≥ 200 mg/dL (≥11.1 mmol/L)]. Normal glucose tolerance was defined as FPG < 100 mg/dL and 2 h-PG < 140 mg/dL. Prediabetes encompassed isolated IFG and IGT, as well as their combination. Prediabetes and T2DM were classified as AGT.

### 4.3. Anthropometric Measurements and Biochemical Assays

Information on maternal age and pre-pregnancy weight were obtained during interviews. The physical examination included an assessment of weight at GDM diagnosis and at the 1-year follow-up. For the biochemical and expression assays, maternal blood samples (20 mL) were collected at the time of GDM diagnosis and 1 year after delivery, in all cases after an overnight fast of at least 8 h. The plasma glucose concentration was measured using the glucose oxidase method. The concentrations of plasma lipids, including TC, TGs, HDL-C, and LDL-C, were analyzed enzymatically using kits from Roche Diagnostics GmbH (Mannheim, Germany). The HbA1C level was measured by a latex-enhanced turbidimetric immunoassay using specific monoclonal antibodies. The concentration of CRP was determined by turbidimetric assay using the cassette COBAS INTEGRA C-Reactive Protein (Latex, Roche Diagnostics GmbH, Mannheim, Germany). FI was measured using an Elecsys Insulin assay (Roche Diagnostics GmbH, Mannheim, Germany). The analyses were conducted using a Cobas Integra automatic chemistry analyzer (Roche Diagnostics GmbH, Basel, Switzerland).

### 4.4. Calculations

Pre-pregnancy or postpartum BMI was computed as self-reported maternal pre-pregnancy or measured postpartum weight in kilograms divided by the square of height in meters. GWG was calculated by subtracting pre-pregnancy weight from the weight measured at GDM diagnosis. Gestational age was calculated according to the date of the last menstrual period and verified by ultrasonography. Insulin resistance and beta-cell function were estimated by the HOMA method, HOMA-IR, and HOMA-B, respectively [56]:

HOMA-IR = [fasting insulin (µU/mL) × fasting glucose (mg/dL)]/405

HOMA-B = [360 × fasting insulin (µU/mL)]/[fasting glucose (mg/dL) − 63]

### 4.5. Blood Collection and Isolation of Blood Leukocytes

As mentioned above, a 20 mL aliquot of blood was withdrawn from each participant at GDM diagnosis and 1-year postpartum: 10 mL for determining biochemical parameters and 10 mL for research purposes. Leukocytes were isolated immediately after blood draws, according to the procedure described previously [14]. In brief, after centrifuging the patient’s blood sample and discarding the plasma, the erythrocytes were lysed using red blood cell lysis buffer erythrocyte lysis buffer (0.5 M NH_4_Cl, 10 mM KHCO_3_, 10 mM EDTA, pH 8.0). The sample was then centrifuged and the supernatant removed, and the pellet containing leukocytes was washed with phosphate-buffered saline (PBS).

### 4.6. RNA Extraction and cDNA Synthesis

Total RNA was extracted from the leukocytes using TriReagent (Sigma-Aldrich, St. Louis, MO, USA) according to the manufacturer’s protocol. RNA quantity and quality were assessed by a BioDrop UV/VIS spectrophotometer (SERVA, Heidelberg, Germany). For the first-strand cDNA synthesis reaction, total RNA (4 μg) was mixed with 200 units of Maxima reverse transcriptase enzyme, 20 units of RiboLock RNase inhibitor, 10 mM of dNTP solution mix, 100 µM of random hexamer and oligo(dT)18 primers, 4 µL of RT buffer (5×), and nuclease-free water, according to the manufacturer’s recommendation (Thermo Scientific, Waltham, MA, USA). The reaction was performed at 50 °C for 30 min for the first-strand cDNA synthesis followed by a 5 min incubation at 85 °C for enzyme deactivation.

### 4.7. mRNA Expression by Quantitative RT-PCR

The relative expression of all the studied genes (*INSR*, *IRS1*, *IRS2*, *PIK3R1*, *SLC2A1*, *SLC2A3,* and *SLC2A4*) was analyzed at the time of GDM diagnosis and 1 year after delivery. For this purpose, the synthesized cDNA was amplified by qPCR using the GoTaq^®^ qPCR Master Mix (2×) reaction kit (Promega, Madison, WI, USA) and the corresponding gene-specific primers, which were designed to amplify transcripts across an exon junction to avoid genomic DNA contamination using the online NCBI Primer-Blast program (https://www.ncbi.nlm.nih.gov/tools/primer-blast, accessed on 15 September 2022) (Table 8) [57]. Reactions were performed in duplicate on a LightCycler 480 II (Roche Diagnostics GmbH, Basel, Switzerland) with initial denaturation at 95 °C for 2 min, followed by 40 cycles of 95 °C for 20 s and 60 °C for 20 s. The target gene expression was normalized to the house-keeping gene *ACTB*, encoding β-actin, and relative expression was determined using the threshold cycle (Ct) value according to the Pfaffl method [58].

### 4.8. Statistical Analysis

For each set of values, data are shown as medians with the first and third quartiles. Between-group differences are shown as fold changes (FC). The comparison between any two groups was performed using a two-sample *t* test (for normally distributed variables) or the Mann–Whitney *U* test (for non-normally distributed variables), and adjusted for maternal age. For follow-up changes in the same group (i.e., pGDM vs. GDM), a paired *t* test or Wilcoxon rank sum test was employed. Nonparametric Spearman correlation analyses were conducted in each group of patients, and the Benjamini–Hochberg (false discovery rate; FDR) procedure was applied [59]. ROC analyses were used to assess the predictive potential of all the variables for postpartum AGT, and optimal cut-off points were indicated by Youden’s index. Logistic regression models were built based on selected variables indicated by the largest AUC in the ROC analysis. Forward and backward stepwise methods were used for building regression models, and the v-fold cross-validation method was applied. The resulting models were assessed by ROC analysis, considering the accuracy in the validation sample as the most important determinant of the models’ quality. The significance level for all the tests was set to α = 0.05. An FDR-adjusted *p*-value < 0.1 (*q*-value) was considered significant.

Data were analyzed using Statistica software, Version 13 (Dell Inc. ^®^, Tulsa, OK, USA), with the analytical extension Zestaw Plus, Version 5.0.85 (Statsoft, Poland).

#### Sample Size Calculation

The size of the NGT and GDM (pGDM) groups was defined based on a preliminary gene expression assay. Calculations were performed using the standard sample size calculator module of the *Statistica* software. A significance level of 95% and a power of 80% for the two-tailed tests (paired *t* test and non-parametric Wilcoxon test) were assumed [60]. For all the genes with substantial expression changes (Cohen’s *d* > 0.8), a sufficient sample size was calculated as no more than 30 patients per group (Table S4).

## Figures and Tables

**Table 1:** Clinical characteristics of the GDM (*n* = 48) and NGT (*n* = 44) groups.

Variable	GDM	NGT	FC	*p*-Value
Maternal age [years]	**32.0 (29.0–35.0)**	**29.0 (26.0–32.0)**	**1.10**	**0.014**
Gestational age at OGTT [week]	28.0 (27.0–29.0)	27.0 (26.0–30.0)	1.04	0.550
Pre-pregnancy BMI [kg/m^2^]	24.9 (22.6–28.1)	24.2 (22.2–26.8)	1.03	0.352
Pregnancy weight [kg]	76.6 (69.3–85.0)	74.1 (65.6–85.8)	1.03	0.219
GWG [kg]	9.0 (5.4–11.3)	8.0 (5.0–12.0)	1.13	0.853
FPG [mg/dL]	**87.0 (81.0–94.0)**	**81.0 (74.5–84.5)**	**1.07**	**<0.001**
1 h-PG [mg/dL]	**182.5 (156.0–192.0)**	**152.0 (129.0–163.0)**	**1.20**	**<0.001**
2 h-PG [mg/dL]	**158.0 (145.0–167.0)**	**139.5 (120.0–147.0)**	**1.13**	**<0.001**
HbA1C [%]	5.0 (4.8–5.3)	5.2 (5.0–5.5)	0.95	0.472
FI [μIU/mL]	**12.4 (7.9–15.8)**	**6.8 (2.6–8.9)**	**1.82**	**<0.001**
HOMA-B	144.2 (116.8–221.9)	124.6 (70.4–174.0)	1.16	0.068
HOMA-IR	**2.6 (1.7–3.8)**	**1.3 (0.5–1.82)**	**1.96**	**<0.001**
TC [mg/dL]	249.0 (224.2–279.0)	271.5 (236.0–296.7)	0.92	0.075
HDL-C [mg/dL]	78.0 (65.1–87.0)	81.7 (70.4–91.1)	0.95	0.241
LDL-C [mg/dL]	134.0 (114.0–159.0)	144.0 (125.0–168.0)	0.93	0.217
TGs [mg/dL]	216.9 (182.1–257.3)	214.00 (179.2–246.8)	1.01	0.746
CRP [mg/dL]	4.1 (2.1–6.1)	3.3 (2.0–6.4)	1.23	0.692

Abbreviations: BMI, body mass index; CRP, C-reactive protein; FI, fasting insulin; FPG, fasting plasma glucose; GWG, gestational weight gain; HDL, high-density lipoprotein; HOMA-B, homeostasis model assessment of β-cell function; HOMA-IR, homeostasis model assessment of insulin resistance; LDL, low-density lipoprotein; PG, plasma glucose; TC, total cholesterol; TGs, triglycerides. Values are given as median with 25% and 75% quartiles. Differences between the groups are expressed as fold change (FC). *p*-value is assessed by Mann–Whitney *U* test. Significant differences between the groups are presented in bold.

**Table 2:** Clinical characteristics of the postpartum GDM group (pGDM, *n* = 48).

Variable	pGDM	FC pGDM vs. GDM	*p*-Value pGDM vs. GDM	FC pGDM vs. NGT	*p*-Value pGDM vs. NGT
Postpartum BMI [kg/m^2^]	24.6 (21.9–30.1)	0.99	0.505	1.02	0.440
FPG [mg/dL]	90.2 (86.0–98.0)	**1.04**	**0.028**	**1.11**	**<0.001**
2 h-PG [mg/dL]	105.5 (92.0–125.0)	**0.67**	**<0.001**	**0.76**	**<0.001**
HbA1C [%]	5.1 (5.0–5.3)	1.03	0.790	0.98	0.792
FI [μIU/mL]	9.5 (5.9–12.4)	**0.77**	**0.001**	**1.40**	**0.005**
HOMA-B	115.4 (89.2–173.6)	**0.69**	**<0.001**	0.93	0.831
HOMA-IR	2.1 (1.2–3.1)	**0.78**	**0.005**	**1.52**	**0.001**
TC [mg/dL]	187.0 (158.0–206.0)	**0.75**	**<0.001**	**0.69**	**<0.001**
HDL-C [mg/dL]	59.0 (51.0–68.0)	**0.76**	**<0.001**	**0.72**	**<0.001**
LDL-C [mg/dL]	97.0 (86.0–132.0)	**0.72**	**<0.001**	**0.67**	**<0.001**
TGs [mg/dL]	91.3 (61.3–114.1)	**0.42**	**<0.001**	**0.43**	**<0.001**
CRP [mg/dL]	1.8 (1.0–3.3)	**0.44**	**<0.001**	**0.55**	**0.002**

Abbreviations as in Table 1. Values are given as median with 25% and 75% quartiles. Differences between the groups are expressed as fold change (FC). *p*-value is assessed by Wilcoxon rank sum test for pGDM vs. GDM and Mann–Whitney *U* test for pGDM vs. NGT. Significant differences between the groups are presented in bold.

**Table 3:** Relative mRNA expression and fold change (FC) of individual genes in leukocytes of women during and after pregnancy.

Group/ Comparison	*SLC2A1*	*SLC2A3*	*SLC2A4*	*PIK3R1*	*INSR*	*IRS1*	*IRS2*
NGT (*n* =44)	0.008	0.108	0.261	0.140	0.240	2.122	0.563
	(0.006–0.010)	(0.025–0.215)	(0.145–0.510)	(0.071–0.377)	(0.013–0.603)	(0.427–4.091)	(0.117–0.903)
GDM (*n* = 48)	0.013	0.432	0.194	1.058	0.355	0.498	0.470
	(0.011–0.017)	(0.313–0.715)	(0.134–0.536)	(0.803–1.442)	(0.065–0.808)	(0.105–1.259)	(0.015–0.970)
pGDM (*n* = 48)	0.019	0.595	0.642	0.673	0.329	0.893	0.577
	(0.014–0.023)	(0.178–0.897)	(0.399–1.194)	(0.510–0.942)	(0.014–0.805)	(0.438–2.069)	(0.053–4.427)
GDM/NGT							
FC	**1.61**	**4.01**	0.75	**7.57**	1.48	**0.23**	0.83
*p*-value	**<0.001**	**<0.001**	0.312	**<0.001**	0.371	**0.046 ^t^**	0.705
pGDM/GDM							
FC	**1.44**	1.38	**3.30**	**0.64**	0.93	1.79	1.23
*p*-value	**0.005**	0.626	**<0.001**	**0.012 ^w^**	0.975	0.221	0.157 ^w^
pGDM/NGT							
FC	**2.32**	**5.51**	**2.46**	**4.81**	1.37	**0.42**	1.02
*p*-value	**<0.001**	**<0.001**	**0.002**	**<0.001**	0.651	**0.030 ^t^**	0.667

Values are given as median with 25% and 75% quartiles. *p*-value is assessed by Mann–Whitney *U* test or *t* test (marked as t) for GDM vs. NGT and pGDM vs. NGT. *p*-value is assessed by paired *t* test or Wilcox rank sum test (marked as w) for pGDM vs. GDM. Significant differences between the groups are presented in bold.

**Table 4:** Spearman’s rank order correlation analysis of the GDM group.

Variable	*SLC2A1*	*SLC2A3*	*SLC2A4*	*PIK3R1*	*INSR*	*IRS1*	*IRS2*
Maternal age [years]	0.20	−0.03	−0.26	0.07	0.00	−0.03	0.03
Pre-pregnancy BMI [kg/m^2^]	−0.08	−0.21	−0.36	0.15	−0.17	−0.36	0.26
Pregnancy weight [kg]	−0.24	−0.17	−0.31	0.04	−0.17	−0.37	0.46 *
GWG [kg]	0.10	0.12	0.19	0.24	−0.05	−0.23	0.17
FPG [mg/dL]	0.26	0.04	−0.27	0.18	0.18	0.13	0.42 *
1 h-PG [mg/dL]	0.06	0.08	−0.12	0.19	0.10	−0.20	0.02
2 h-PG [mg/dL]	0.06	−0.41	−0.12	0.14	−0.15	−0.03	0.18
HbA1C [%]	0.25	0.06	−0.13	0.16	−0.10	−0.20	0.14
FI [μIU/mL]	−0.02	−0.24	−0.31	−0.18	−0.26	−0.36	0.44 *
HOMA-B	−0.16	−0.25	−0.29	−0.33	−0.30	−0.54 *	0.15
HOMA-IR	−0.01	−0.18	−0.28	−0.19	−0.21	−0.22	0.48 **
TC [mg/dL]	−0.23	−0.02	0.29	−0.02	0.04	−0.15	−0.16
HDL-C [mg/dL]	−0.43	−0.05	0.16	−0.07	0.00	0.14	−0.20
LDL-C [mg/dL]	0.08	0.09	0.15	0.00	0.01	−0.07	−0.13
TGs [mg/dL]	−0.02	−0.31	−0.20	0.06	−0.06	−0.43	0.20
CRP [mg/dL]	−0.06	0.03	0.11	−0.23	0.09	−0.12	0.21

Abbreviations as in Table 1. Data are shown as *rho* correlation coefficient. * *p* < 0.05; ** *p* < 0.01. Significant correlations after FDR correction (*q* < 0.1) are presented in bold.

**Table 5:** Spearman’s rank order correlation analysis of the pGDM group.

Variable	*SLC2A1*	*SLC2A3*	*SLC2A4*	*PIK3R1*	*INSR*	*IRS1*	*IRS2*
Maternal age [years]	−0.10	−0.16	−0.20	0.05	0.01	0.22	−0.03
Pre-pregnancy BMI [kg/m^2^]	−0.36	−0.03	0.03	−0.23	0.22	−0.07	0.19
Postpartum BMI [kg/m^2^]	0.00	−0.24	−0.31	0.22	−0.06	−0.36	0.21
FPG [mg/dL]	0.13	−0.44	−0.52 *	0.58 *	0.05	0.08	0.29
2 h OGTT [mg/dL]	−0.21	0.29	−0.26	−0.05	−0.02	−0.08	0.32
HbA1C [%]	−0.05	−0.37	−0.14	0.33	0.02	−0.18	0.21
FI [μIU/mL]	0.01	−0.36	−0.43	0.35	−0.25	−0.28	0.32
HOMA-B	0.03	−0.05	0.02	−0.09	−0.11	−0.23	0.09
HOMA-IR	−0.06	−0.44	−0.48 **	0.39	−0.25	−0.19	0.26
TC [mg/dL]	0.04	0.04	−0.15	0.07	0.08	−0.16	−0.12
HDL-C [mg/dL]	−0.10	0.38	0.49	−0.13	0.21	0.52 *	−0.28
LDL-C [mg/dL]	0.17	−0.19	−0.24	0.16	−0.10	−0.35	−0.05
TGs [mg/dL]	−0.11	0.06	−0.17	−0.38	0.07	−0.28	0.13
CRP [mg/dL]	−0.34	0.17	0.08	−0.31	−0.09	−0.43	0.25

Abbreviations as in Table 1. Data are shown as *rho* correlation coefficient. * *p* < 0.05; ** *p* < 0.01. Significant correlations after FDR correction (*q* < 0.1) are presented in bold.

**Table 6:** Predictive potential of gene expression and anthropometric/biochemical parameters of patients during pregnancy for postpartum abnormal glucose tolerance (pAGT), assessed by ROC analysis.

Variable	AUC	SE	95% CI	*p*-Value	Cut-Point	Youden’s Index	Sensitivity *	Specificity *
*SLC2A1*	0.63	0.15	0.32–0.93	0.416	0.01	0.35	0.75	0.60
*SLC2A3*	0.65	0.13	0.40–0.90	0.234	0.38	0.53	1.00	0.53
*SLC2A4*	0.58	0.13	0.32–0.83	0.562	0.21	0.40	1.00	0.40
*PIK3R1*	0.62	0.16	0.31–0.92	0.456	0.99	0.35	0.50	0.85
*INSR*	**0.77**	**0.09**	**0.59–0.94**	**0.003**	**0.66**	**0.54**	**0.70**	**0.84**
*IRS1*	0.71	0.12	0.46–0.95	0.097	0.55	0.55	1.00	0.55
*IRS2*	0.63	0.11	0.42–0.84	0.238	0.05	0.32	0.90	0.42
Maternal age [years]	0.47	0.09	0.29–0.65	0.737	26.00	0.09	1.00	0.09
Pre-pregnancy BMI [kg/m^2^]	0.66	0.10	0.46–0.86	0.114	28.91	0.48	0.57	0.91
Pregnancy weight [kg]	**0.71**	**0.09**	**0.52–0.89**	**0.030**	**82.90**	**0.49**	**0.69**	**0.79**
GWG [kg]	0.47	0.10	0.28–0.66	0.732	9.60	0.17	0.46	0.71
FPG [mg/dL]	0.65	0.09	0.48–0.82	0.094	101.00	0.31	0.43	0.88
1 h-PG [mg/dL]	0.63	0.10	0.44–0.83	0.185	178.00	0.37	0.83	0.54
2 h-PG [mg/dL]	0.43	0.10	0.23–0.63	0.518	167.00	0.12	0.36	0.77
HbA1C [%]	0.62	0.10	0.42–0.81	0.241	5.42	0.28	0.39	0.90
FI [μIU/mL]	0.51	0.11	0.29–0.72	0.965	25.30	0.28	0.31	0.97
HOMA-B	0.43	0.10	0.24–0.63	0.494	191.52	0.16	0.54	0.63
HOMA-IR	0.53	0.11	0.32–0.75	0.757	6.37	0.28	0.31	0.97
TC [mg/dL]	0.44	0.09	0.26–0.63	0.543	260.00	0.12	0.46	0.66
HDL-C [mg/dL]	0.64	0.10	0.44–0.84	0.171	61.10	0.37	0.46	0.91
LDL-C [mg/dL]	0.50	0.09	0.32–0.69	0.990	157.00	0.14	0.39	0.75
TGs [mg/dL]	0.47	0.10	0.28–0.66	0.785	217.70	0.18	0.62	0.56
CRP [mg/dL]	0.57	0.09	0.39–0.75	0.436	3.75	0.22	0.69	0.53

Abbreviations: AUC, area under curve; SE, standard error; CI, confidence interval. The remaining abbreviations as in Table 1. AUCs significantly larger than 0.5 (*p* < 0.05) are presented in bold. * represents cut-points.

**Table 7:** Logistic regression model for prediction of postpartum AGT.

Source	*B (SE)*	Wald *χ*2	*p*-Value	OR (95% CI)
Intercept	−9.44 (3.96)	5.67	0.017	0.00 (0.00–0.19)
Pregnancy *INSR* expression	2.45 (1.09)	5.02	0.025	11.61 (1.36–99.16)
Pregnancy body weight [kg]	0.09 (0.04)	4.32	0.038	1.09 (1.01–1.19)

Abbreviations: AGT, abnormal glucose tolerance; CI, confidence interval; OR, odds ratio; SE, standard error.

**Table 8:** PCR primer list.

GenBank	Gene	Description		Sequence 5′ → 3′	Product Length [bp]
NM_006516.4	SLC2A1	Solute carrier family 2 member 1	F R	CTTCACTGTCGTGTCGCTGT TGAGTATGGCACAACCCGC	95
NM_006931.3	SLC2A3	Solute carrier family 2 member 3	F R	TGGAGAAAACTTGCTGCTGAGA TCAGAGCTGGGGTGACCTTCT	141
NM_001042.3	SLC2A4	Solute carrier family 2 member 4	F R	CCTGCCAGAAAGAGTCTGAA GCTTCCGCTTCTCATCCTT	85
NM_181523.3 NM_181504.4 NM_181524.2 NM_001242466.2	PIK3R1	Phosphoinositide-3-kinase regulatory subunit 1	F R	GGAAGCGAGATGGCACTTTT ACAATGCTTTACTTCGCCGTC	92
NM_000208.4 NM_001079817.3	INSR	Insulin receptor	F R	CAAGCACTTCGCTCTGGAAC ACGTCTAAATAGTCTGTCACGTAG	144
NM_005544.3	IRS1	Insulin receptor substrate 1	F R	TCAAGTGAGGATTTAAGCGCC TTAGGTCTTCATTCTGCTGTGA	100
NM_003749.3	IRS2	Insulin receptor substrate 2	F R	ACCATCGTGAAAGAGTGAAGAT ACACAGTCATTGCTCAGATCCA	142
NM_001101.5	ACTB	Actin beta	FR	GCACAGAGCCTCGCCTT GTTGTCGACGACGAGCG	93

Abbreviations: bp, base pairs; F, forward; R, reverse.

## Data Availability

The datasets used and analyzed during the current study are available from the corresponding authors on reasonable request.

## Supplementary Materials

The following supporting information can be downloaded at: https://www.mdpi.com/article/10.3390/ijms252313094/s1.
