# Supplementary material for: Assessment of Changes in the Expression of Genes Involved in Insulin Signaling and Glucose Transport in Leukocytes of Women with Gestational Diabetes During Pregnancy and in the Postpartum Period
Source: Int J Mol Sci. 2024 Dec 5;25(23):13094. doi: 10.3390/ijms252313094 (PMC11641716; doi:10.3390/ijms252313094)
Supplement: Supplementary file 1 [file ijms-25-13094-s001.zip › ijms-3309105-supplementary.pdf]

Table S1. General linear models (ANCOVA) for differences in chosen variables between the GDM and NGT groups with adjustment for maternal age

Variable	Source	SS	DF	MS	F	p
Fasting insulin	Intercept	**490.193**	**1**	**490.1934**	**7.53635**	**0.007446**
	Age	61.147	1	61.1466	0.94008	0.335143
	Group	**926.628**	**1**	**926.6278**	**14.24619**	**0.000304**
	Error	5268.554	81	65.0439		
HOMA-IR	Intercept	**28.9714**	**1**	**28.97141**	**6.01668**	**0.016319**
	Age	4.8215	1	4.82151	1.00131	0.319973
	Group	**67.6723**	**1**	**67.67234**	**14.05395**	**0.000332**
	Error	390.0299	81	4.81518		

Table S2. General linear models (ANCOVA) for gene expression differences between the GDM and NGT groups with adjustment for maternal age

Gene	Source	SS	DF	MS	F	p
SLCA1	Intercept	**0.000168**	**1**	**0.000168**	**9.49448**	**0.003937**
	Age	0.000007	1	0.000007	0.37055	0.546529
	Group	**0.000201**	**1**	**0.000201**	**11.32716**	**0.001827**
	Error	0.000637	36	0.000018		
SLCA3	Intercept	0.066109	1	0.066109	0.82723	0.369125
	Age	0.004036	1	0.004036	0.05050	0.823461
	Group	**0.877382**	**1**	**0.877382**	**10.97869**	**0.002108**
	Error	2.877005	36	0.079917		
SLCA4	Intercept	**1.086941**	**1**	**1.086941**	**9.790888**	**0.003468**
	Age	**0.515675**	**1**	**0.515675**	**4.645065**	**0.037905**
	Group	0.012438	1	0.012438	0.112037	0.739779
	Error	3.996562	36	0.111016		
PIK3R1	Intercept	0.258414	1	0.258414	2.28718	0.140261
	Age	0.009153	1	0.009153	0.08101	0.777767
	Group	**2.118038**	**1**	**2.118038**	**18.74637**	**0.000138**
	Error	3.615485	32	0.112984		
INSR	Intercept	0.00615	1	0.006152	0.025395	0.873992
	Age	0.21632	1	0.216324	0.893040	0.348943
	Group	0.19563	1	0.195635	0.807627	0.372888
	Error	12.83839	53	0.242234		
IRS1	Intercept	282.32	1	282.320	0.425395	0.517575
	Age	39.45	1	39.452	0.059445	0.808483
	Group	1894.06	1	1894.059	2.853933	0.098069
	Error	29864.98	45	663.666		
IRS2	Intercept	1.9679	1	1.967882	0.730991	0.396484
	Age	0.1539	1	0.153889	0.057164	0.811976
	Group	2.0659	1	2.065916	0.767407	0.385049
	Error	139.9879	52	2.692075		

Table S3. Spearman's rank order correlation analysis in the GDM group

**Variable**	***SLC2A1***	***SLC2A3***	***SLC2A4***	***PIK3R1***	***INSR***	***IRS1***	***IRS2***
Maternal age [years]	-0.24	0.06	-0.48	0.00	0.39	0.22	0.25
Pre-pregnancy BMI [kg/m2]	-0.01	-0.07	0.19	-0.01	0.02	0.03	0.26
Pregnancy weight [kg]	-0.04	0.08	0.13	-0.04	-0.08	0.02	0.22
GWG [kg]	-0.08	0.33	-0.38	-0.01	-0.52	-0.45	-0.38
FPG [mg/dL]	-0.06	0.00	0.00	0.18	0.31	0.28	0.16
1h-PG[mg/dL]	0.29	0.15	0.09	0.16	0.22	0.23	-0.01
2h-PG [mg/dL]	0.23	0.29	0.04	0.33	-0.04	-0.33	-0.30
HbA1C [%]	-0.17	0.01	0.01	-0.22	-0.09	-0.26	-0.11
FI [μIU/ml]	-0.32	-0.16	-0.09	-0.10	0.00	-0.01	0.07
HOMA-B	-0.37	-0.30	-0.05	-0.27	0.14	0.16	0.27
HOMA-IR	-0.31	-0.18	-0.07	-0.09	0.05	-0.03	0.05
TC [mg/dL]	-0.18	0.21	-0.18	-0.60	0.08	0.08	0.18
HDL-C [mg/dL]	-0.17	0.04	-0.38	0.45	-0.06	0.04	-0.03
LDL-C [mg/dL]	0.02	0.14	-0.05	-0.53	0.11	0.08	0.15
TGs [mg/dL]	-0.45	-0.11	-0.17	-0.84	-0.13	-0.29	-0.17
CRP [mg/dL]	0.19	0.20	0.02	0.13	0.14	0.22	0.28

Abbreviations as in Table 1. Data are shown as rho correlation coefficient and p-values. All the correlations were insignificant after a FDR correction ($q > 0.1$)

Table S4. Sample size for each group determined to detect changes in gene expression with 80% power at α= 0.05 for two-sided tests based on preliminary data (NGT, *n* =20; GDM and pGDM, *n* =20)

Gene	NGT	GDM	pGDM	GDM vs NGT Cohen's *d*	GDM vs NGT sample size	pGDM vs GDM sample size
SLC2A1	0.009(0.004)	0.014(0.004)	0.018(0.007)	1.24	14	20
SLC2A3	0.167(0.204)	0.511(0.316)	0.572(0.392)	1.30	13	742
SLC2A4	0.417(0.388)	0.340(0.332)	0.798(0.465)	0.21	365	12
PIK3R1	0.247(0.305)	0.775(0.341)	1.198(0.744)	1.63	10	30
INSR	0.382(0.404)	0.527(0.557)	0.522(0.606)	0.30	187	227482
IRS1	2.863(3.329)	15.511(39.474)	11.335(37.375)	0.45	84	1105
IRS2	0.675(0.687)	1.049(2.141)	7.231(17.961)	0.24	300	71

Data shown as mean and standard deviation
